# Preferences of the Saudi Population in Breaking Bad Medical News: A Regional Study

**DOI:** 10.7759/cureus.19525

**Published:** 2021-11-13

**Authors:** Mohammed Basheikh

**Affiliations:** 1 Department of Medicine, King Abdulaziz University Faculty of Medicine, Jeddah, SAU

**Keywords:** diagnosis disclosure, relative, preference, patient, breaking bad news

## Abstract

Objective

To explore the preference among the Saudi population regarding breaking bad news (BBN) for the participant cases and their relatives and to determine the associated sociodemographic factors.

Method

A cross-sectional study was conducted among patients and companions attending inpatient and outpatient clinics of a tertiary care hospital in Western Saudi Arabia from 15 Jan to 30 May 2015. A six-item scale was designed to assess preference regarding diagnosis disclosure in three hypothetical conditions including chronic disease, incurable disease, and cancer if the participant or a close relative is concerned, separately. A BBN preference score (BBN-PS) was computed (range=0-6), with a higher score indicating a greater preference to disclose the diagnosis. Eventual motivations for diagnosis disclosure or withholding were explored.

Result

Five hundred participants were included; 56.0% were females and 55.0% were aged between 18 and 25 years. Preference to be informed with one’s diagnosis varied between 81.8% for incurable disease and 94.2% for chronic disease with complications. Preference to inform a relative with their diagnosis ranged between 69.0% for incurable disease and 86.8% for chronic disease with complications. Preference for diagnosis withholding was lower among participants of the younger age category (38.2% vs 51.2% or higher, p=0.002), with higher education (42.4% vs 60.8%, p=0.001), and working or studying in the medical field (39.7% vs 51.9%, p=0.006), compared to their counterparts, respectively. The most common motivations toward diagnosis disclosure preference were to enable the concerned person participate in their therapeutic decision (36.4%) and cope with the disease (27.4%); while preference toward diagnosis withholding was most commonly motivated by apprehensions regarding the psychological and social impact of the diagnosis (61.6%).

Conclusion

A non-negligible proportion of individuals prefer concealing a diagnosis of cancer or incurable disease to a relative, with an inter-generational disparity showing a shift to diagnosis disclosure in the young generations. There is an unmet need for evidence-based protocols for BBN based on a comprehensive assessment of patients’ expectations and needs, considering their cultural and religious values as well as the specific sociodemographic and clinical factors.

## Introduction

“Bad News” was defined by Robert Buckman, a British oncology registrar in 1984, as “any news that drastically and negatively alters the patient’s view of his or her future”[1]. From a healthcare perspective, when the bad news is conveyed poorly, the patient may have adverse reactions such as denial of further treatment, decreased medication compliance, and emotional distress. These may severely compromise the quality of care and impact the prognosis and quality of life [2,3]. Therefore, delivering such news to the patient requires a set of communication skills, aiming to help the patients accept their medical condition and cope with the novel situation while reinforcing the trust relationship. Among strategies advocated and widely accepted in breaking bad news (BBN) to patients is the SPIKES protocol. The Acronym SPIKES stands for Setting up, Perception, Invitation, Knowledge, and Emotions, Strategy & Summary [4]. Physicians are recommended to be trained for such strategies especially in settings with a high likelihood of exposure to the relevant situations [2,5].

Several patient-related factors can interfere with the BBN process requiring specific adaptation by physicians to the particular case of the patient and his or her environment. Among these factors are the patient preference and values and the family environment. While Western societies strongly advocate for patient’s autonomy and the right to be informed of their medical condition, regardless of the severity and prognosis, Eastern societies provide a central role to the family’s point of view in the patient’s management and the related decision-making process [6,7]. This may reflect in complex care conditions, where families may express their preference to withhold the diagnosis or prognosis information from the patient, in a protective attitude. In some cases, the patients may prefer to be uninformed about their medical condition, treatment, and prognosis, or can be selective regarding the content to be disclosed or the setting or manner in which they would like to be informed [8,9]. Such situations expose the healthcare provider to a major ethical conflict, notably the balance between the patient’s right to know and family interest in protecting the patient from emotional harm, in addition to the eventual legal conflicts with families in case the bad news impacts the patient's wellbeing [10-12].

Consequently, it is important to understand the preference for BBN in addition to the related sociocultural factors, in considering the supportive family and social environment as well as the value system that may help the patients cope with the disease and preserve their quality of life. In this study, we aimed to explore the preference among the Saudi population regarding BBN for the participant cases and their relatives and to determine the associated sociodemographic factors.

## Materials and methods

Methods

Design and Setting

A cross-sectional study was conducted at outpatient clinics of King Abdulaziz University (KAU) Hospital, Jeddah, Saudi Arabia, between 15 January and 30 May 2015. The study was approved by the institutional review board of KAU.

Participants

Adult patients and companions (age 18 years and above) who visited one of the participating clinics during the study period were included. Patients having cancer or terminal illnesses or their companions, and those with mental disorders or communication difficulties were excluded.

Sampling

The sample size was calculated to detect an unknown proportion (P=50%) of participants with either preference regarding BBN, with 0.05 type I error, 0.20 type II error, and 95% CI among an infinite population. The sample size was calculated as 377, which was increased to 500, anticipating a 25% invalid or incomplete participation. A convenience sampling technique was used to enroll all voluntary participants until reaching the target sample size.

Data Collection

A semi-structured questionnaire was developed for this study. The questionnaire used in the present study was designed to assess the preference in BBN of each participant, both as a patient and a relative of a patient. It was used to collect the following data: 1) sociodemographic data including gender, age, nationality, educational level, and job sector (healthcare vs non-healthcare); 2) a six-item dichotomous scale (I would inform them/prefer being informed versus I would not inform them/prefer not being informed) to measure preference for BBN regarding a hypothetical diagnosis, for own and a close relative, of a chronic disease, an incurable disease, and cancer; 3) eventual motivations for withholding bad medical news; and 4) eventual motivations for not withholding the bad medical news. Both parts 3 and 4 used a list of predefined options in addition to a free option (other, specify).

The questionnaire underwent face and content validity. Subsequently, it was edited in both English and Arabic languages and was administered with a face-to-face interview, using the preferred language of the participant. A team of trained medical students was formed for data collection, which took place during working weekdays between 8 AM and 5 PM. All participants signed an informed consent form approved by the Research Ethics Committee of KAU.

Statistical Analysis

Data was entered, coded, and analyzed using the Statistical Package for Social Sciences version 21.0 for Windows (SPSS Inc., IBM, NY, USA). Descriptive statistics were used to summarize the pattern of answers to the different questionnaire sections. Pearson’s correlation coefficient was calculated to analyze the bivariate correlation between the BBN preference (BNNP) scale, and the internal consistency of the scale was analyzed by calculating Cronbach’s alpha. A BBNP score was calculated as the sum of the six items’ score, by attributing one point to each response favoring disclosure of the news. Thus, the BBNP ranged from 0 to 6, with higher score indicating preference to disclose the diagnosis to the concerned person (self or relative) and lower score indicating the likelihood of withholding the bad medical news. Chi-square test was used to analyze factors associated with the likelihood to withhold the bad medical news using two different cutoffs BBNP < 6 and BBNP < 5. A P value of <0.05 was considered to reject the null hypothesis.

## Results

 

Participants’ characteristics

Five hundred participants were interviewed and were included in our study. 56.0% were females and 55.0% were aged 18-25 years. Other characteristics included higher educational level (bachelor or higher education, 80.6%) and 48.4% were working or studying in the medical field (Table 1).

**Table 1:** Questionnaire about preferences to break bad medical news

If you were diagnosed with cancer, do you prefer being informed?	Yes, I would like to know	No, I do not want to know
If a relative is diagnosed with cancer, do you prefer telling them?	Yes, I will tell him/her	No, I will not inform him/her
If you were diagnosed with an incurable disease, do you prefer being informed?	Yes, I would like to know	No, I do not want to know
If a relative is diagnosed with an incurable disease, do you prefer telling them?	Yes, I will tell him/her	No, I will not inform him/her
If you were diagnosed with a chronic disease that has complications, do you prefer being informed?	Yes, I would like to know	No, I do not want to know
If a relative is afflicted with a chronic disease that has complications, do you prefer telling them?	Yes, I will tell him/her	No, I will not inform him/her

Preference in BBN

**Figure 1:**
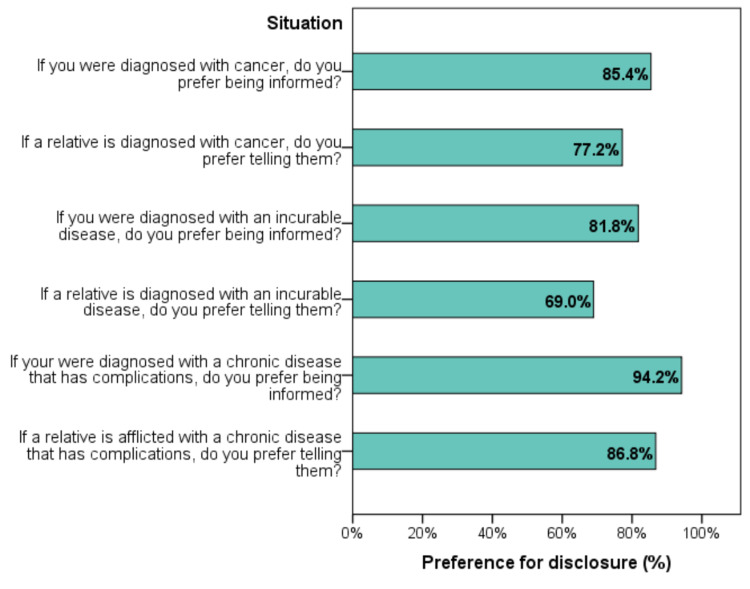
Preference in breaking bad news

Preference to be informed with one’s own diagnosis varied between 81.8% for incurable disease and 94.2% for chronic disease with complications. Preference to inform a relative with their diagnosis ranged between 69.0% for incurable disease and 86.8% for chronic disease with complications (Figure 1).

Internal consistency of the BBNP questionnaire

**Figure 2:**
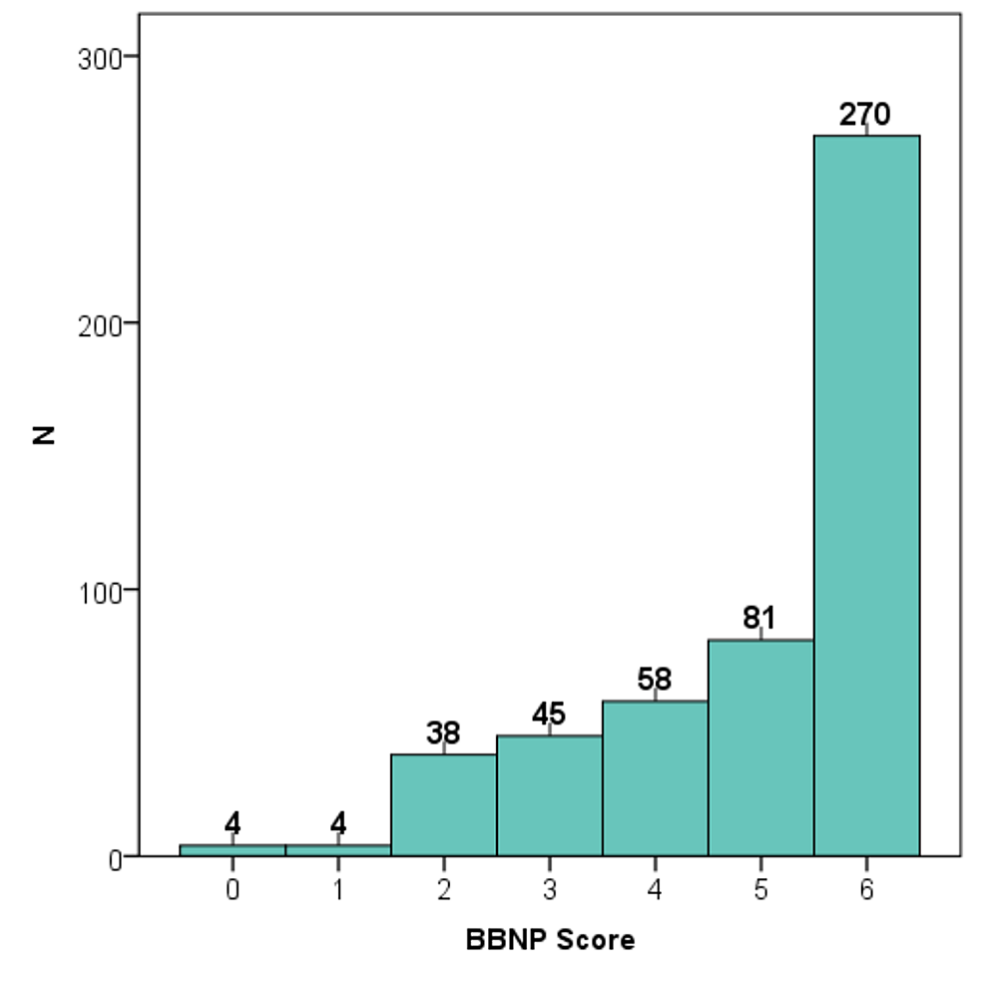
Internal consistency of the BBNP questionnaire BBNP: breaking bad news preferences

The internal consistency of the BBNP questionnaire showed Cronbach’s alpha=0.707, indicating the reliability of the answers. Inter-item correlations were generally weak, with Pearson’s correlation coefficients ranging between 0.000 (Item 2 x Item 3) and 0.700 (Item 4 x Item 6) (Table 2). A BBNP score (range 0-6) was calculated, showing a mean=4.94 (SD=1.42); and the histogram distribution of the score showed that 46% of the participants would be favorable to withhold bad news in at least one situation (BBNP score≤5), and 29.8% would be favorable in at least two situations (BBNP score≤4) (Figure 2).

**Table 2:** Participants’ characteristics (N=500)

Parameter	Category	Frequency	Percentage
Gender	Male	220	44.0
	Female	280	56.0
Age category	18-25	275	55.0
	26-35	121	24.2
	36-45	55	11.0
	45-60	42	8.4
	61 and above	7	1.4
Nationality	Saudi	405	81.0
	Non-Saudi	95	19.0
Educational level	Uneducated	3	0.6
	Elementary	5	1.0
	Middle school	14	2.8
	Secondary	75	15.0
	Bachelor	363	72.6
	Post-graduate	40	8.0
Work or study in the medical field	No	258	51.6
	Yes	242	48.4

Factors associated with preference in BBN

BBNP score was significantly higher among the younger age category (P<0.001) and participants working or studying in the medical field (P=0.041) compared to their counterparts. Further, by using the cut-off level (BBNP≤ 5), the likelihood of withholding the bad medical news was lower among participants of the younger age category (38.2% vs 51.2% or higher, P=0.002), with higher education (42.4% vs 60.8%, P=0.001), and working or studying in the medical field (39.7% vs 51.9%, P=0.006), compared to their counterparts. By using the cut-off (BBNP ≤4), the likelihood of withholding the bad medical news was similar between the age categories (26-35 years and 18-25 years) and the education level. The difference between these two categories was not statistically significant (Tables 3-4).

**Table 3:** Correlation between the different questionnaire items

	If a relative is afflicted with a chronic disease that has complications, do you prefer telling them?	If you were diagnosed with a chronic disease that has complications, do you prefer being informed?	If a relative is diagnosed with an incurable disease, do you prefer telling them?	If you were diagnosed with an incurable disease, do you prefer being informed?	If a relative is diagnosed with cancer, do you prefer telling them?	If you were diagnosed with cancer, do you prefer being informed?
If a relative is afflicted with a chronic disease that has complications, do you prefer telling them?	-	0.080	0.135	0.076	0.182	-0.027
If you were diagnosed with a chronic disease that has complications, do you prefer being informed?	0.080	-	0.000	0.437	0.008	0.455
If a relative is diagnosed with an incurable disease, do you prefer telling them?	0.135	0.000	-	0.367	0.677	0.323
If you were diagnosed with an incurable disease, do you prefer being informed?	0.076	0.437	0.367	-	0.337	0.700
If a relative is diagnosed with cancer, do you prefer telling them?	0.182	0.008	0.677	0.337	-	0.396
If you were diagnosed with cancer, do you prefer being informed?	-0.027	0.455	0.323	0.700	0.396	-

**Table 4:** Factors associated with preference in breaking bad news BBNP: breaking bad news preferences

Factor	Category	BBNP score			Preference level 1 (BBNP ≤5)		Preference level 2 (BBNP ≤4)	
		Mean	SD	P-value	%	P-value	%	P-value
Gender	Male	4.90	1.42		48.6		30.5	
	Female	4.98	1.43	0.500	43.9	0.294	29.3	0.777
Age category	18-25	5.15	1.37		38.2		22.5	
	26-35	4.92	1.38		51.2		28.9	
	36-45	4.25	1.64		60.0		52.7	
	45-60	4.62	1.36		61.9		47.6	
	61+	4.86	1.21	<0.001*	57.1	0.002*	42.9	<0.001*
Nationality	Saudi	4.96	1.42		44.9		29.9	
	Non-Saudi	4.87	1.45	0.593	50.5	0.325	29.5	0.938
Educational level	Low	4.78	1.46		60.8		28.9	
	High	4.98	1.41	0.217	42.4	0.001*	30.0	0.823
Work or study in the medical field	No	4.82	1.43		51.9		34.9	
	Yes	5.08	1.41	0.041*	39.7	0.006*	24.4	0.010*

Motivations for each preference

The most commonly specified motivations for not withholding the bad medical news were to enable the concerned person participate in their therapeutic decision (36.4%) and cope with the disease (27.4%), other unspecified reasons (17.2%), followed by enabling the support of religion and spirituality (11.2%). The most common motivation for withholding the bad medical news was fear of the psychological and social impact of the diagnosis on the patient (61.6%), followed by the desire to prevent impacting patient’s confidence in the physician’s therapeutic decision (10.2%), while 20.8% did not provide a response (Table 5).

**Table 5:** Participant-reported critical motivation for each preference in breaking bad medical news (N=500)

Preference	Motivation	Frequency	Percentage
Not withhold bad medical news	To participate in the therapeutic decision	182	36.4
	To follow up on health condition	37	7.4
	To enable coping with the disease	137	27.4
	To strengthen religious and spiritual dimension	56	11.2
	Other reasons	86	17.2
	No response	2	0.4
Withhold bad medical news	No response	104	20.8
	Fear of the psychological and social impact	308	61.6
	Issue with the patient's confidence in the physician’s therapeutic decision	51	10.2
	Other social motivations	35	7.0
	Other—not specified	2	0.4

## Discussion

The present study provided insight regarding the preference of BBN among a representative sample of patients and visitors of a referral tertiary care center in Western Saudi Arabia. Although the majority of participants displayed preferring diagnosis disclosure in all explored situations, almost half of them (46%) were favorable to withhold bad diagnosis in at least one care situation. Preference regarding BBN varied significantly depending on the sociodemographic profile including age, education, and job sector. Participants of younger age, higher education, and or healthcare sector jobs were more favorable to disclose the diagnosis compared to their counterparts. Apprehensions regarding the impact of the diagnosis disclosure on the patient’s psychological health or confidence in the medical decision were the most significant motivations for withholding bad medical news; while expectations toward patient’s participation in care, coping and seeking religious and spiritual support were reported as the major drivers for diagnosis disclosure.

Preference in BBN in Muslim countries is scarcely documented, especially related to disclosure of diagnosis [13]. An interesting Saudi study, which involved 402 mothers of hospitalized children with no previous experience of BBN, explored the mothers’ preference related to BBN about their children’s diagnosis. Findings showed that 17% of mothers would prefer BBN process to involve the husband first, and the latter would decide news transmission to them; and 24% of the participants agreed that husband-mediated bad news disclosure would enable minimizing their trauma. Regarding the BBN content, 16% expressed preference for a brief over detailed content and 42% favorized gradual communication of the details. Further, 56% of the mothers opted for the presence of a supportive person from the relatives during BBN process [14]. Although the latter study outlined a different aspect of the issue with reference to the present study. Cross-interpretation of the findings from these two studies highlights the presence of a significant percentage of individuals (~15-20%) who prefer not to directly being exposed to bad news disclosure by the medical teams and may prefer remaining uninformed. Comparable to the present study findings, an Indian study involving cancer patients showed that 72% agreed to be informed of their cancer diagnosis and majority were favorable to involve their relatives in the BBN process [15].

Beyond the “disclosure versus non-disclosure” issue that was explored in the present study, preference regarding the disclosure process is another relevant dimension that warrants further investigation and would provide valuable practical indications in BBN optimization in accordance with patients’ values and expectations. International data shows that a high percentage of cancer patients are unsatisfied with the process of their cancer diagnosis disclosure to them directly, reflecting a disparity between patients’ preferences and the implemented BBN approach. Some studies assessed the efficiency of established recommendations, such as the SPIKES protocol, which was initially evaluated and validated for the United States and subsequently recommended in several other countries [4,16,17]. A study from the UK showed that approximately 60% of the oncology patients were satisfied with the disclosure of their cancer diagnosis to them, highlighting the importance of the doctor’s empathy and positive attitude in enhancing their satisfaction. Further, the study showed the prominent role of the doctor’s competency, expertise, and communication skills in enhancing patient’s satisfaction with BBN. On the other hand, patients displayed lower consideration for other supportive aspects including emotional support and involvement of relatives, and majority opted for a collaborative or an active role in the therapeutic decision [18]. In Malaysia, patients exhibited higher consideration for the doctor’s attitude and communication skills while delivering the information, as well as the content of the news [19]. Another study from Germany reported even lower satisfaction figures, with only 46% of cancer patients being entirely satisfied with the BBN process. The satisfaction levels were inversely correlated with the subsequent emotional state of the patient as indicated by the occurrence of anxio-depressive and sleeplessness disorders. Further analysis showed the critical impact of physician-patient communication in optimizing BBN process, highlighting the frequent patients’ preference for an adapted process and accurate understanding of their specific needs [20]. These studies led to amendments of the established protocols to meet the actual patients’ preference; thus indicating the unmet need for evidence-based protocols for BBN based on comprehensive assessment of patients’ expectations, considering the cultural background and specific sociodemographic and clinical factors. Additionally, the efficient implementation of such protocols should consider and enable further evaluations notably patient’s satisfaction.

Findings from the dimension related to diagnosis disclosure to relatives showed preference to withhold bad medical news among approximately 23% and 31% in case of cancer and incurable disease, respectively. This probably denotes a protective attitude, notably to prevent eventual psychological and social impact of such diagnoses, which was legitimized by approximately 62% of the participants. The next most common apprehension regarding diagnosis disclosure to the concerned relative was the impact on the physician-patient relationship, which was reported by approximately 10% of the participants. The role and involvement of patient’s relatives in care practice is common in Islamic societies and has several benefits to the patient. However, there is a lack of clear guidelines to regulate the level of involvement and define the limits of the responsibilities [7]. By comparison, a Togolese population-based study explored preference about diagnosis disclosure using a fictive scenario involving a fictive relative represented by a cognitively competent 70-year-old female person in various situations. From a set of five predefined options, 52% preferred not disclosing the diagnosis to the patient, while 26% preferred telling the truth to relatives and only 1% opted to tell the truth always. Interestingly, the religion factor showed a higher proportion of Muslim participants (64%) opting for diagnosis non-disclosure to the patient, compared with their Christian counterparts (37%) [21]. Beyond this cultural effect, and in a broader concept, healthcare providers and institutions should consider implementing a family centered care approach as part of the organizational culture. Such an approach has the advantage of strengthening the care relationship while enhancing satisfaction among patients, their families, and care providers [12].

The present study showed that preference regarding BBN was associated with several sociodemographic factors. A generation effect was observed, where younger participants displayed more openness toward diagnosis disclosure compared with the older ones. Comparable observations were reported among cancer patients, indicating negative relationship between the patient’s age and the preference to obtain elaborate medical information about the diagnosis and prognosis [20]. This is also consistent with other data showing propensity of older patients toward passive role during BBN process, compared with younger ones who were more oriented toward a collaborative role [18]. Another factor that showed a statistically significant association with a preference for BBN was the educational level. Expectedly, highly educated participants were more inclined to diagnosis disclosure. This is consistent with data showing inclination to passive role among poorly educated cancer patients in the UK with reference to highly educated patients who preferred the collaborative role [18]. In contrast, highly educated Togolese individuals were more favorable toward diagnosis non-disclosure to the patient, compared with poorly educated patients who were more inclined to disclose the diagnosis to relatives rather than directly to the patient [21]. These observations further demonstrate the need for an evidence-based approach to establish recommendations for BBN that are adapted to the patient’s specificities, including cultural background, clinical condition, and further specific needs. Such an approach can only be enabled by extensive investigations of patients’ preferences in various settings and populations along with accurate analysis of the associated factors.

The present study assessed one aspect of BBN, using hypothetical situations, which might differ from real situations. Further, the study involved individuals attending one care center limiting the generalizability of the findings.

## Conclusions

In conclusion, it is found that a non-negligible proportion of individuals prefer concealing a diagnosis of cancer or incurable disease to a relative, denoting a protective attitude. Such attitude is largely legitimized to prevent eventual psychological and social impact on the concerned person. A generation effect was observed in terms of BBN preference, showing a shift to diagnosis disclosure in the young generations. There is an unmet need for evidence-based protocols for BBN based on comprehensive assessment of patients’ expectations and needs, considering their cultural and religious values as well as the specific sociodemographic and clinical factors. The efficient implementation of such protocols should enable further evaluations via the assessment of patient’s feedback besides additional supportive measures that may be necessary for some specific subpopulations or settings.
